# Supplementary material for: Types, design, implementation, and evaluation of nutrition interventions in older people in Africa: A scoping review protocol
Source: PLoS One. 2024 Nov 8;19(11):e0313036. doi: 10.1371/journal.pone.0313036 (PMC11548761; doi:10.1371/journal.pone.0313036)
Supplement: Attachment — Submitted filename: PLOS ONE Peer review response 2024.09.24.docx [file pone.0313036.s003.docx]

**Authors’ response to peer review feedback**

We thank the editor and reviewer for their feedback on our protocol. Our point-by-point response to the feedback is in blue font below.

Reviewer #1: The protocol addresses an important research question for the region- very well done on this.

Thank you.

I have a few things the authors may wish to think more about.

Review questions

The use of the phrase "recommended" in the question raises some concerns, how does this fit into the inclusion criteria in the end - recommended by who? I do not see this reflected in the PICO

Thank you for this comment and upon reflection we agree that is does not fit with the PICO criteria and would lead to inconsistencies in data reported as recommended/implied interventions may not have other relevant data such as design and implementation. We have therefore deleted recommended from the research question to read:

*What types of nutrition interventions have been implemented for older adults living in Africa?*

1) Include a justification for including older persons in long term care facilities and provide more descriptive details on what type of facilities are being considered here and the purpose for which older persons are in these facilities.

Thank you for this comment. Given the scoping nature of this review and likely limited literature on the topic we aim to collate all relevant literature available regardless of where interventions are delivered. We did not have a justification to exclude older adults in long-term facilities and a systematic review on rehabilitation interventions after hip fracture has found that residency in nursing homes as one of the main drivers of exclusion from such interventions contributing to inequity and limited generalisability of such interventions, <https://doi.org/10.1093/ageing/afz031>. We have revised the statement to reflect this under the population section in Table 2, which now reads:

*Older adults who are community dwelling or residing in any long-term care facilities such as nursing homes, retirement centres. Inclusion of older adults in long-term facilities promotes equity and generalisation of findings [23].*

2) You may wish to specify what kind of qualitative studies are being targeted for inclusion, is it descriptive, process evaluations or??

Thank you for this suggestion. We will include any relevant qualitative study and have noted this in the revised statement on Study design in Table 2:

Any study design including randomised trials, quasi experiments, qualitative studies such as implementation studies, process evaluations, case studies with relevant data to the research questions

3) The data analysis/synthesis section is too brief and unclear. Please provide more details on how review questions will be answered at this level.

Thank you for this suggestion. We have now added Table 3 which provides more details on analysis for each review question, see below:

| **Intervention characteristic** | **Review questions (RQs)** | **Analysis/synthesis** |
| --- | --- | --- |
| **Types** | 1. What types of nutrition interventions have been implemented for older adults living in Africa? | Descriptions of different intervention types, diseases or outcomes targeted, and purpose of intervention *i.e.,* prevention or treatment |
|  | 2. What are the components of implemented interventions? | Descriptions of components of interventions *e.g.,* for education interventions, written information, or oral group counselling |
| **Design** | 3. Who and what should nutrition interventions target? | **Who:** Descriptions of factors associated with nutrition and nutrition outcomes, categorised into four nutritional influences drawing from the framework by Story et al[24]  **What:** pooled prevalences of nutrition outcomes (*e.g.,* malnutrition, obesity, weight loss) from meta-analyses |
|  | 4. How have interventions been designed? | Descriptions of evidence base supporting intervention formative work, theory of change behavioural framework supporting intervention and end-user engagement during intervention (co-)development |
| **Implementation** | 5. How have interventions been (or can be) implemented? | Descriptions of implementation characteristics including setting (*e.g*., community, hospital); intervention duration and dose; approach (e.g., individual versus group) and mode of delivery (e.g., peers, health care workers), complex or stand alone intervention |
|  | 6. What implementation approaches influence intervention uptake, acceptability, feasibility, and effectiveness? | Quantitative and qualitative data from process evaluations on implementation approaches (e.g., incentivisation) and contextual factors that influence uptake, acceptability, feasibility, fidelity and effectiveness |
|  | 7. What is the cost and sustainability of implemented interventions? | Costs of interventions, or components of intervention (e.g., direct health costs, patient out-of-pocket costs), and descriptions on their sustainability and health economic modelling of future intervention / scale-up. |
| **Evaluation** | 8. How have the interventions been evaluated? | Description of study designs (e.g., randomised controlled trials, quasi-experimental, process evaluation, cost evaluation), primary and secondary outcomes used to evaluate interventions and their delivery, consideration of risks of bias. |

4) How will the workshop participants be identified and later contacted and by who? The details of this process are lacking.

Thank you for this suggestion. Workshop participants will be identified through professional and local networks based on a stakeholder mapping performed as part of a wider study, <https://wellcomeopenresearch.org/articles/8-261>. They will be contacted by in-country project coordinators and invited for the workshop. We have revised the text on pages 10 to read:

*Eight to ten stakeholders will be identified through professional and local networks after stakeholder mapping and will focus on local nutrition experts and older adults, and/or community-based representatives, who will be potential intervention end-users. In-country project coordinators will invite the stakeholders for the consultation.*
